# Clinical Trials of Thermosensitive Nanomaterials: An Overview

**DOI:** 10.3390/nano9020191

**Published:** 2019-02-02

**Authors:** Stefania Nardecchia, Paola Sánchez-Moreno, Juan de Vicente, Juan A. Marchal, Houria Boulaiz

**Affiliations:** 1 Department of Applied Physics, Faculty of Sciences, University of Granada, C/Fuentenueva s/n, 18071 Granada, Spain; stefania@ugr.es (S.N.); jvicente@ugr.es (J.d.V.); 2 Excellence Research Unit “Modeling Nature” (MNat), University of Granada, 18016 Granada, Spain; jmarchal@ugr.es; 3 Nanobiointeractions & Nanodiagnostics, Istituto Italiano di Tecnologia, Via Morego, 30, 16163 Genova, Italy; paola.sanchez@iit.it; 4 Department of Human Anatomy and Embryology, University of Granada, 18016 Granada, Spain; 5 Biopathology and Medicine Regenerative Institute (IBIMER), University of Granada, 18016 Granada, Spain; 6 Biosanitary Institute of Granada (ibs.GRANADA), SAS-Universidad de Granada, 18016 Granada, Spain

**Keywords:** thermosensitive nanomaterials, USPIO, magnetic nanoparticles, Ferumoxytol, gold nanoparticles, ThermoDox

## Abstract

Currently, we are facing increasing demand to develop efficient systems for the detection and treatment of diseases that can realistically improve distinct aspects of healthcare in our society. Sensitive nanomaterials that respond to environmental stimuli can play an important role in this task. In this manuscript, we review the clinical trials carried out to date on thermosensitive nanomaterials, including all those clinical trials in hybrid nanomaterials that respond to other stimuli (e.g., magnetic, infrared radiation, and ultrasound). Specifically, we discuss their use in diagnosis and treatment of different diseases. At present, none of the existing trials focused on diagnosis take advantage of the thermosensitive characteristics of these nanoparticles. Indeed, almost all clinical trials consulted explore the use of Ferumoxytol as a current imaging test enhancer. However, the thermal property is being further exploited in the field of disease treatment, especially for the delivery of antitumor drugs. In this regard, ThermoDox®, based on lysolipid thermally sensitive liposome technology to encapsulate doxorubicin (DOX), is the flagship drug. In this review, we have evidenced the discrepancy existing between the number of published papers in thermosensitive nanomaterials and their clinical use, which could be due to the relative novelty of this area of research; more time is needed to validate it through clinical trials. We have no doubt that in the coming years there will be an explosion of clinical trials related to thermosensitive nanomaterials that will surely help to improve current treatments and, above all, will impact on patients’ quality of life and life expectancy.

## 1. Introduction

Nowadays, there is a real need to seek out more efficient systems for the diagnosis and treatment of many diseases and hence achieve better overall health in our society. Sensitive nanomaterials that can respond to exact stimuli are part of an important strategy in many biomedical fields like drug delivery, biosensing, and biomaterials [1,2] These materials can be functionalized to respond to temperature, pH, light, electric field, magnetic field, radiofrequency and ultrasound, amongst many others [3,4]. Specifically, thermosensitive nanomaterials are promising in disease treatment and diagnosis due to their capacity to aim at pre-selected sites when simulated in a certain temperature range [5]. Amid disparities of diversified biomedical applications, thermosensitive nanomaterials have remarkable features, which make them strong candidates for use in medical applications such as drug delivery, diagnostic devices, and thermal therapy. 

The field of thermal therapy has grown exponentially in recent years. Indeed, several studies have been registered using traditional hyperthermia, in combination with chemotherapy and/or radiation therapy, for the elimination of many types of tumors [6,7,8]. In magnetic hyperthermia, which has reduced side effects, tumor cells receive heat through the use of magnetic nanoparticles (MNPs) and an alternating magnetic field (AMF) [9]. AMF heating promotes deep tumor penetration and temperature regulation [10]. MNP-based thermal therapy has remarkable advantages over traditional thermal therapies such as (i) innocuous penetration of frequencies produced by MNPs, (ii) homogenous heat generation, (iii) the possibility of inducing antitumoral immunity, and (iv) the development of a multi-modality device providing thermal therapy and magnetic resonance imaging (MRI) [11]. Among different clinical therapies, magnetic fluid hyperthermia (MFH)-based thermotherapy has received great interest as an antitumor strategy, wherein ultrasmall superparamagnetic iron oxide nanoparticles (USPIOs) are principally used to induce localized therapeutic heat (reaching 42−45 °C) inside the tumors [12]. Also, the heat generated from specific nanoparticles (i.e., gold nanoparticles (GNPs)) can be utilized to eradicate/damage cancerous cells by photothermal therapy (PTT) [13]. When a laser is focused on a tissue, the photons are absorbed by the cellular and intercellular areas and their energies are converted into heat, inducing cellular death. Unlike traditional hyperthermia, PTT is produced in the area directly surrounding nanoparticles, and local temperatures can rise, in very short time scales, by tens or hundreds of degrees Celsius above physiological temperature. This would help to reduce the side effects of the antitumor treatments because the treatment can be directed to the targeted tissue.

Controlling the application of thermal energy to living tissues is a great challenge, which is driving the development of many devices and treatment techniques, both at preclinical and clinical levels [14]. The trend is to improve non-invasive monitoring methods in contrast to existing techniques, such as tissue biopsies, that are based on destructive/invasive methods.

Non-invasive procedures like positron emission tomography (PET) and MRI lack the specificity to be a feasible alternative to cell tracing. Single photon emission computed tomography, even though it permits non-invasive tracking of in vivo bio-dissemination of radiotracers at picomolar concentrations, has several disadvantages (e.g., limited spatial resolution, lack of anatomical details for reference, etc.) that make it difficult to target the exact location of lesions. The combination of different imaging modalities using multimodal probes can be of great interest in molecular imaging [15]. This synergistic combination provides improved visualization of biological targets, and obtains information on all aspects of cell or tissue structure and function, which is hard to obtain by a single imaging modality [16]. 

New devices based on thermal nanoparticles combined with multi-modality imaging would simplify non-invasive monitoring of treatments, since they could allow simultaneous dynamic imaging of both structure and function and directly provide valuable information on pharmacokinetics and drug metabolism. Moreover, nanomaterials can provide information on the biological state of cells in addition to their physical location [17]. 

Until now, many types of nanomaterials have been used to build thermosensitive nanosystems for therapy and diagnosis, such as inorganic nanoparticles, carbonaceous materials, and liposomal formulations [5,18,19].

### 1.1. Inorganic Nanoparticles

Among the extensive variety of inorganic nanoparticles reported in the literature, MNPs, USPIOs, and GNPs experience increased relevance as thermosensitive nanomaterials for biomedicine [20,21]. 

MNPs are known to be very promising for biomedical treatments [22]. In particular, iron oxide nanoparticles (IONs) possess an inherit elemental composition that makes them biocompatible and degradable by nature [23]. IONs can be produced in very small sizes (<40 nm), with uniform size distribution, superparamagnetic nature, possibility of functionalization with different molecules (drug molecules, fluorescent compounds [24,25,26]), solubility in water along with a high magnetic moment. These are simply some of the characteristics that make these materials attractive for many applications, e.g., hyperthermia and magnetic resonance enhancement. The combination of magnetic and other properties in one single nanoparticle can create a path to new multifunctional nanomaterials that display multimodal properties [27,28]. For example, externally applied magnetic fields can be used to address some specific targeted functionalization such as the controlled release of drugs in desired areas, gathering the nanocomposites in a specific region for diagnosis or treatment and, finally, eradicating them at the end of the whole procedure [29,30]. It is also important to point out that multimodal MNPs can also open the access to the monitoring of all the possible steps included in the treatments by taking advantage of numerous imaging procedures like MRI and computed tomography (CT) [31,32].

Among the various MNPs, the USPIOs are very useful for biomedical applications [33,34]. The USPIOs are magnetite (Fe_3_O_4_) and/or maghemite (γ-Fe_2_O_3_) particles, usually coated with different surface coating molecules, such as citrate, polymers, and silica. This hydrophilic coating helps the water dispersibility and improves the colloidal stability and biocompatibility of the USPIOs [35,36]. 

In the family of nanoparticles, GNPs are unlike any others, displaying some unique optical properties, where the origin is due to an electromagnetic phenomenon called surface plasmon resonance (SPR) [37]. SPR depends on several characteristics of GNPs like, for example, their size, composition, shape, etc. [38]. The control of SPR effect on GNPs has been shown to lead to important bioimaging applications and/or photothermal agents [39,40]. Last but not least, there is another quite interesting property of GNPs, which is the heat generation after absorbing infrared light. After excitation by the light source, the nanoparticles face the possibility to dissipate the heat into their surroundings, melt, or even cause structural damage. This unique property has been exploited for PTT applications [41,42]. 

### 1.2. Carbon-Based Nanomaterials 

Other types of inorganic materials actively investigated in biomedical applications are carbon-based nanomaterials (CBNs) [43]. One popular two-dimensional (2D) CBN is a derivative of graphite, termed graphene, a single layer of sp^2^-bonded carbon atoms. The main characteristics of these materials are high mechanical strength and excellent thermal and electrical conductivity. Graphene in its oxidized form, graphene oxide (GO), exhibits oxygen atoms on the edges and basal planes of its structure. The functional groups with oxygen atoms (epoxy, hydroxyl, carboxylic and carbonyl, etc.) turn out to be ideal binding sites for different molecules, such as polymers and biological molecules [44], and increases its hydrophilicity in comparison to graphene. It has been reported that GO is also non-toxic and biocompatible at low concentrations [45]. Another interesting feature is a high photothermal conversion capability in the near-infrared (NIR) wavelength region. Thus, the combination of strong absorption and heat transfer can be used to kill tumor cells directly [46]. In addition, incorporating GO nanosheets into thermosensitive hydrogels may reveal some novel and interesting applications. The main advantage of a thermosensitive hydrogel is that it is a viscous liquid at low temperature, which allows easy handling, incorporation of cells or biomolecules, and finally injection in a minimally invasive manner. Importantly, shape stability at the injection site is warranted by their gelation at body temperature. Incorporating CBNs improves their fracture strength and, in some cases, the elastic modulus as well, which is a requirement for load-bearing applications. The kinetics of the temperature response can be tuned by the quality and quantity of the filler, thereby broadening their possible applications as sensors and drug delivery systems.

### 1.3. Liposomal Formulations

In addition to inorganic materials, thermosensitive liposomes (TSLs) are also interesting instruments for drug delivery [24,47]. Liposomes are spherical vesicles composed of a membrane bilayer, usually constituted of phospholipids and an aqueous core [48]. Conventional liposomes are approved for use as delivery systems for clinical use in oncology [49]. However, the liposomes present poor penetration and a limited drug release factor in the tumor area. Otherwise, upon administration, circulating TSLs are activated locally under conditions of mild hyperthermia by increasing the temperature (to 40–41 °C) using an external heat source [50]. The increase of the temperature causes compositional changes in the liposomal membrane, creating openings that permit the release of the encapsulated drug. These thermo-devices favor the specific release of the largest amount of cytotoxic agent to a heat-treated tumor site, limiting the injury to the surrounding normal tissue [51]. To overcome these limitations, TSLs have been developed as new controlled drug release systems [52]. ThermoDox®, a TSL containing DOX, is the only TSL formulation to arrive at drug development. In the few last years, research has focused on solving the limitations associated with the ThermoDox® formulation, such as poor circulation lifetime or the membrane permeability of DOX for different diseases [53]. In this regard, Lindner et al. developed new TSLs with prolonged liposome circulation time for mild hyperthermia, and showed an increased plasma half-life for the loaded drug with *t*_1/2_ = 9.6 h in hamsters and *t*_1/2_ = 5.0 h in rats dependent on changes in composition of TSLs [54]. Limmer et al. showed an increase in plasma half-life of 0.53 to 2.59 h for gemcitabine (GEM) when the size of TSLs was increased [55]. Furthermore, the development of TSLs loaded with non-membrane permeable drugs (e.g., cisplatin (HTLC) [56], GEM, oxaliplatin [57]) allows their use for different diseases that cannot be treated with DOX (e.g., breast, ovarian, cervical, gastric cancers). Moreover, new applications of the liposomal formulations will open new biomedical possibilities. [58,59]. For example, the synergy of TSLs and magnetic resonance-guided drug delivery will open the possibility to target desired injured sites and online monitoring at the same time [60,61]. This is just one example of the extraordinary true theranostic nature of thermosensitive liposomes, that is the ability to combine specific targeted therapy based on specific targeted diagnosis. In this regard, Rizzitelli et al. [62] published a preclinical study describing a novel protocol for the DOX release from TSLs monitored by MRI in a breast cancer mouse model. In addition, DI-TSLs (DOX and indocyanine green (ICG) loaded TSLs) capable of responding to NIR were manufactured. This theranostic system allows the control of the release of drugs from TSLs (PTT for hyperthermia drug release) and uses fluorescence of the same ICG to continuously monitor the drug-release, biodistribution and antitumor efficacy of the DI-TSL system in the body [63]. Recently, a microfluidic chip has been developed capable of adjusting the temperature rise for the drug control release by TSL. This system allows procedures such as cytometric analysis and AFM scanning, as well as presenting small size, biocompatibility and low cost [64]. Table 1 lists some of the recent publications related to the development of new TSLs formulations and new applications.

**Figure 1:**
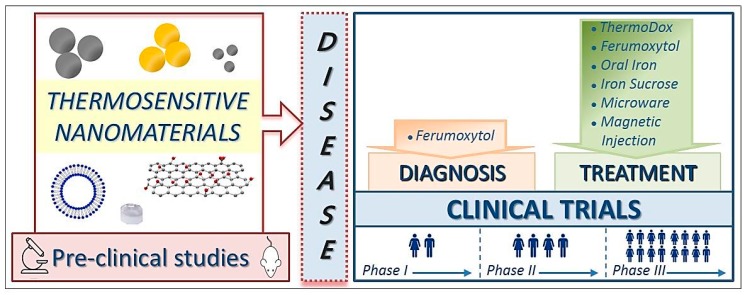
Illustration of the uses of thermosensitive nanomaterials.

Next, we will focus on a review of the clinical trials carried out to date with thermosensitive nanomaterials including all those clinical trials in which the nanomaterials of interest are hybrid and also respond to other stimuli (e.g., magnetic, infrared radiation and ultrasound). Specifically, we discuss their use in both diagnosis and treatment of different diseases without forgetting those already approved by Food and Drug Administration (FDA) for clinical use (Figure 1).

## 2. Clinical Trials and Marketed Thermosensitive Nanomedicines

**Figure 2:**
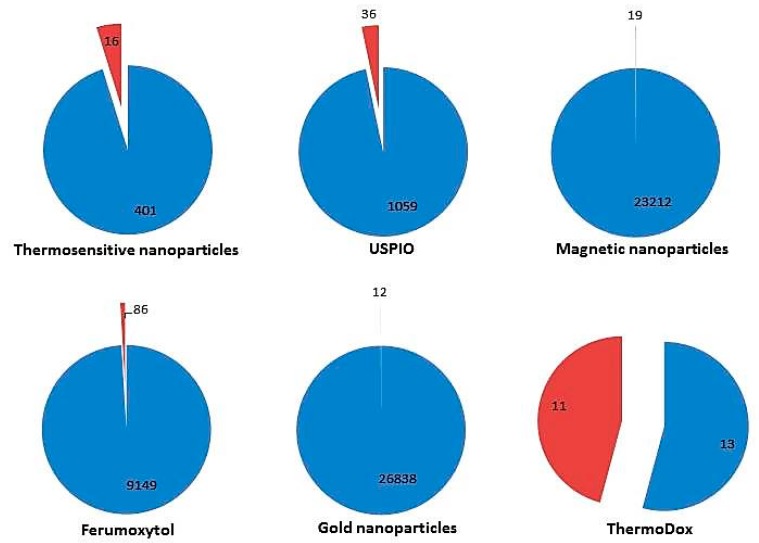
Number of publications (blue) versus clinical trials (red) related to thermosensitive nanomaterials, USPIOs, MNPs, GNPs, Ferumoxytol and ThermoDox®. Data reported here reflect manuscripts available through PubMed database and clinicaltrials.gov up to November 2018.

Several clinical trials are currently being conducted or have been completed in a wide spectrum of pathologies (https://clinicaltrials.gov/). Its use is focused on both treatment and diagnosis of different diseases and some of them have been approved by regulatory bodies. We have used the terms “Thermosensitive”, “USPIO”, “Magnetic nanoparticles”, “Ferumoxytol”, “gold nanoparticles” or “ThermoDox” as keywords when looking for this information. When conducting our search, we have observed that a discrepancy exists between the number of published papers in thermosensitive nanomaterials and the number of nanomaterials in clinical use (Figure 2).

### 2.1. Thermosensitive Nanomaterials for Disease Diagnosis

Until now, the thermosensitive nanomaterials used in the diagnosis of different pathologies do not take advantage of the thermal characteristics of these materials. Indeed, among all thermosensitive nanoparticles, the majority of clinical trials have been registered with USPIOs as contrast agents and only one clinical trial uses MNPs to separate mature sperm from immature ones. However, clinical trials have begun to be recorded in order to investigate the safety of novel nanoparticles such as carbon black and graphene nanoparticles [69] (Table 2). 

#### 2.1.1. Ferumoxytol as a Current Imaging Test Enhancer

USPIOs attracted extensive research efforts for cellular imaging applications and their clinical research has been recently updated by Wang and Idee [70]. Ferumoxytol, an USPIO system developed as an MRI contrast agent in 2000, is better known as a therapeutic agent for the treatment of iron deficiency anemia (IDA) in the setting of chronic kidney disease (CKD). Nevertheless, in recent years, ferumoxytol has regained interest as an agent for MRI enhancement and most of the clinical trials carried out on nanothermal materials focus on their use in the diagnosis of several pathologies. Ferumoxytol, has many characteristics that make it an attractive candidate to be a good contrast agent for vascular and perfusion-weighted MRI (i) easy administration through a rapid bolus, (ii) long intravascular half-life (14–15 h), (iii) less limited by idiosyncratic and allergic reactions in comparison to other USPIOs; (iv) provides a strong T1- and T2-signal on MRI and is taken up by cells in bone marrow. Moreover, ferumoxytol is an iron-based agent that can be an alternative to gadolinium-based contrast agents (GBCAs) in patients with compromised renal function since it does not cause nephrogenic systemic fibrosis [71]. It has been shown that ferumoxytol is finally taken up by macrophages/the reticuloendothelial system in the spleen, liver, and lymph nodes. This uptake mechanism is being used as a novel imaging technique for tumors, vascular lesions, and lymph nodes. Currently, many clinical trials are being conducted to determine its efficacy and safety as an intravenous contrast agent in MRI. 

a. Cancer Diagnosis

In the field of oncology, there are currently several investigations that aim to use ferumoxytol-enhanced MRI to diagnose primary and metastatic tumors. USPIOs do not directly identify the tumoral cells, they help to improve the resolution of the MRI image, which makes it easier to make a differential diagnosis that indirectly, and together with the results obtained through other tests such as steady-state blood volume mapping, indicates the state of the lesion. Although visualization of the lesion with USPIOs is generally similar to GBCAs, differences in improvement patterns, based on the breakdown of the blood-brain barrier marker, can help the differential diagnosis [72]. Parenchymal improvement is best seen in the delayed phase, 24 h after the injection of USPIOs as the extravasation of large molecules is slow. USPIOs improve the delimitation of the edges and allows the evaluation of the internal morphology of the lesion as it has been reflected in several MRI sequences weighted in T1 used clinically. The decrease in the signal in the T2-weighted images in the delayed phase can indicate great local USPIO levels or retention in tumor-associated macrophages. The low T1 and T2 improvement together can help differentiate tumor necrosis (extracellular iron) from solid tumor (intracellular iron). Perfusion MRI and steady-state blood volume mapping can also enhance tumor classification by recognizing the most malignant area for surgical selection and monitoring of therapy [73]. There are currently several clinical trials that aim to use ferumoxytol-enhanced MRI to diagnose primary and metastatic tumors. This is the case with the phase II trial study that will analyze the effectiveness of ferumoxytol, in comparison to gadolinium, in measuring tumors in patients undergoing treatment for brain tumors or other tumors that have metastasized to the brain [74]. In addition, another clinical trial is underway to determine by MRI if ferumoxytol can provide more data to understand the spread of head and neck squamous cell carcinoma and melanoma [75], breast cancer, and prostate cancer [76].

Moreover, another clinical trial focuses on the analysis of how well ferumoxytol-enhanced MRI works in imaging lymph node metastases in patients with different types of cancers such as esophageal cancer [77], advanced rectal cancer [77,78], prostate [79], bladder, and kidney cancers [80], breast cancer [81], and brain neoplasm in both adults and children [82,83,84,85]. 

In addition, ferumoxytol-enhanced MRI can serve not only in the detection of cancer cells but also in the differentiation between malign and infectious diseases. In fact, Macrophages play an important role in the pathogenesis of inflammation. After human intravenous (IV) injection, superparamagnetic iron oxide nanoparticles (SPIOs) arrive at inflammation sites where their small size of (10–30 nm) enables them to leak through permeable capillaries (5–100 nm) into inflamed tissues. Then they are phagocytosed by macrophages displaying prolonged T2 and T2* effects on contrast-enhanced MR images in macrophage-infiltrated tissues [73]. Seyfer et al. [86] found that abscesses (infectious masses) could be differentiated from viable tumor because they displayed a smaller contrast-to-noise ratio than neoplasia when imaged using a T2*-weighted MRI sequence with SPIOs. Twenty-four hours after the USPIO-injection, no changes were observed in VX2 carcinomas, whereas a mean reduction of the contrast-to-noise ratio (CNR) of approximately 90% was noticed in abscesses. In addition to that, necrotic tumors can also be differentiated from viable tumors and from abscesses as they present mixed behavior after USPIO injection. On histopathologic examination, abscess and necrotic parts of the tumor were found to include iron-containing monocytes demonstrating that the reduction in CNR was caused by USPIO-tagged monocytes. In this context, researchers from Stanford University School of Medicine conducted a pilot trial to analyze whether ferumoxytol-enhanced MRI can be used in the differentiation between bone sarcomas and osteomyelitis [87]. Furthermore, ferumoxytol-enhanced MRI can serve also to assess response to therapy since it has been seen that after treatment of brain tumor with chemoradiotherapy or radiation, augmented edema and contrast improvement on MRI may either represent (i) tumor progression since the increase of the tumor mass indicates the failure of the ongoing treatment, or (ii) pseudoprogression, which results in a subacute inflammatory reaction caused by the treatment without underlying tumor [88]. Being able to differentiate between these two procedures is a key element for patient survival. In this sense, some pilot trials study the effectiveness of ferumoxytol-MRI in assessing response to pembrolizumab in patients with tumors on the brain originating from melanoma and glioblastoma metastasis [89], in patients with glioblastoma multiforme receiving temozolomide and radiation therapy [90] and in patients with high grade glioma treated with a bevacizumab and dexamethasone [91]. Diagnostic procedures, such as ferumoxytol MRI, may help measure a patient’s response to antitumor drug treatment. 

b. Non-tumor Pathology Diagnosis

Furthermore, ferumoxytol is also being studied for its applicability as a contrast agent for MRI of non-tumor pathologies such as myocardial Infarction [92], Hereditary Hemorrhagic Telangiectasia [93], Type 1A diabetes [94,95], Renal Transplant Rejection [96] and Osteonecrosis [97]. A randomized phase II trial studies how well gadolinium and ferumoxytol MRI works in diagnosing patients with abnormalities in the Central Nervous System (CNS) [98]. Determining the extent of inflammation associated with pathologies in the CNS may be useful both for diagnosis, prognosis, and monitoring the treatment response of current and future immunotherapies. In this sense, researchers investigate the safety of ferumoxytol as a contrast agent to be used in the identification of neuroinflammation in multiple sclerosis (MS) patients [99]. For that, they want to (i) investigate its safety as determined by a lack of long-term signal change in healthy volunteers (HV) and people with MS; (ii) investigate if this drug improvement can be detected in MS lesions on 7-tesla (T) MRI; and (iii) analyze the special and temporal enhancement patterns of ferumoxytol compared to gadolinium contrast and gradient-echo imaging in MS lesions. A similar trial was carried out on human patients with epilepsy [100].

Human Immunodeficiency Virus (HIV)-associated neurocognitive disorders (HAND) have a significant impact on morbidity and quality of life of patients. HAND remains a chronic issue despite effective combination antiretroviral therapy. It is believed that Monocytes/macrophages play a critical role in the pathogenesis of this pathology. Neuroimaging HIV research has not focused on assessing Monocytes/macrophages-mediated inflammation in the brain. Hence, clinical trials are being conducted to find out if ferumoxytol-based imaging can identify ongoing inflammation due to perivascular monocytes/macrophages, which are thought to represent a key pathological correlate of HAND [101,102]. 

On the other hand, ferumoxytol can be used to enhance Magnetic Resonance Angiography (MRA). Conventional vascular imaging techniques are frequently contraindicated in patients with CKD due to their relative invasiveness and risk. Computed tomography angiography (CTA) requires nephrotoxic iodinated contrast and radiation that can cause a deterioration of renal function and even cause the need for a dialysis. GBCAs for MRA have been related with rare nephrogenic systemic fibrosis disease. Alternative methods of imaging also have drawbacks such as (i) increased risk of complications from conventional invasive catheter angiography, (ii) MRA without contrast permits visualization of smaller arteries but is less precise for larger vascular structures and (iii) ultrasound is frequently not suitable for evaluation of the deep vessels of the abdomen and pelvis. In this sense, a ferumoxytol-enhanced MRA phase 4 clinical trial is being undertaken [103]. Other clinical trials for coronary artery disease, peripheral arterial disease [104] and cerebrovascular disorders [105] are being carried out.

#### 2.1.2. Magnetic Nanoparticles for Sperm Sorting

MNPs are also used in the field of fertility. In fact, a recent clinical trial was initiated in September 2018 and aims to achieve sperm separation by MNPs for Intracytoplasmic sperm microinjection (ICSI) cycles with teratozoospermia and women of over 35 years of age [106]. Women over 35 years old are likely to suffer from impaired oocyte repair capacity. Teratozoospermia is a condition that reflects morphological affection of sperm. These spermatozoa would add an extra burden to the oocyte after ICSI. The hypothesis of this research is whether the selection of mature sperm by a MNPs protocol would provide a more competent sperm to a likely affected oocyte that would improve the ICSI results.

### 2.2. Thermosensitive Nanomaterials for Disease Treatment

Due to its characteristics, thermosensitive materials such as USPIOs, GNPs and lyso-thermosensitive liposomal formulations could be new therapeutic tools whose applicability is being demonstrated through various clinical trials (Table 3).

#### 2.2.1. Iron Deficiency Anemia in Patients with Chronic Kidney Disease

**Figure 3:**
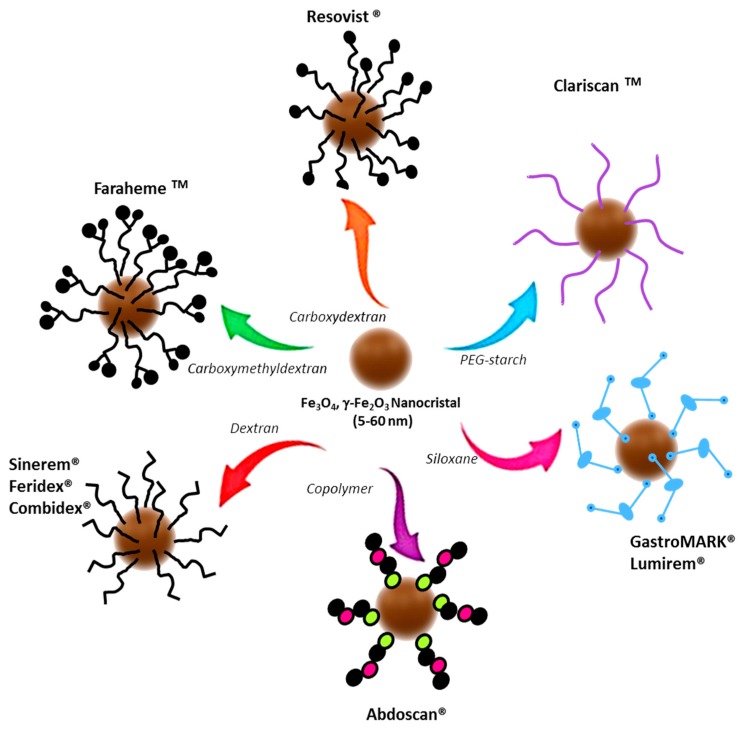
USPIOs clinically approved or in clinical trials.

Many formulations of USPIOs have already been approved by different countries for use in patients with IDA in CKD [118]. These formulations vary according to their coatings, which enhance the superparamagnetic properties of the nanoparticles in a different manner (Figure 3). The recommended treatment is intravenous (IV) iron administration for hemodialysis patients and either oral or IV iron for patients in CKD stages 1–5 who are anemic [119]. Currently, the efforts of researchers are focused on conducting comparative studies to find out which formulation is the best in terms of efficacy and side effects and which route of administration (IV or oral) is safer [120,121]. 

A meta-analysis of small randomized trials reported the efficacy of oral compared to IV on hemoglobin response in CKD patients not on dialysis. In the short-term, one to three months, trials (five of the six trials reported in this meta-analysis) informed that the mean increase in hemoglobin with IV was 0.31 g/dL compared to oral iron. However, in a longer trial (six months), the authors observed a mean decline in hemoglobin of 0.52 g/dL associated with IV administration. The long-term safety of these modes of supplemental iron administration cannot be assessed because the duration of most clinical trials was short [118].

Recently, several clinical trials have been registered comparing ferumoxytol with other superparamagnetic iron formulations. Ferumoxytol (Feraheme™, AMAG Pharma Inc., Lexington, KY, USA) was approved for the treatment of IDA in adults with CKD in the United States and Canada (Feraheme injection) [122] together with the European Union and Switzerland (Rienso, withdrawn in 2015 for commercial reasons) [123]. Different to most other IV iron preparations, a complete cycle of ferumoxytol (1.02 g) requires only two IV injections of 510 mg administered at a rate of up to 1 mL/s (30 mg/mL) between three and eight days apart. The two doses of ferumoxytol may have advantages for both patients and health professionals. In this context, the results of a clinical trial that examined rates of hypersensitivity reactions (HSRs) with IV iron formulations used to treat IDA have been published. In this multicenter, double-blind and randomized clinical trial, researchers compared the safety, and efficiency of ferumoxytol versus ferric carboxymaltose (FCM), aiming at the incidence of moderate-to-severe HSRs, including anaphylaxis, or moderate-to-severe hypotension as the primary end point and the incidence of moderate-to-severe HSRs, including anaphylaxis, serious cardiovascular events, and death as the secondary end point. Ferumoxytol was not inferior to FCM for both the primary and secondary composite safety end points, with an equivalent efficacy in increasing hemoglobin despite a lower dose. However, with FCM treatment severe hypophosphatemia was observed at higher rates [124].

Currently, a phase IV trial of repeated doses of ferumoxytol in comparison to iron sucrose for the treatment of IDA in patients with CKD on hemodialysis has been completed. The objectives of this study were to compare the efficacy and safety of repeat doses of IV ferumoxytol with IV iron sucrose for the treatment of IDA in subjects with hemodialysis-dependent CKD [125]. In 2014, the authors published their prelaminar results that indicate that ferumoxytol and iron sucrose show similar effectiveness and side effect rates [119].

#### 2.2.2. Cardiovascular Diseases

In the field of cardiology, some clinical trials have been registered. Plasmonic nanophotothermal therapy is arousing great interest for atherosclerosis treatment. In fact, clinical trials are being carried out, with the objective of analyzing the feasibility of nanoburning to eliminate and reverse the plaque, especially in combination with stem cell technologies to achieve functional restoration of the vessel wall [115,126]. Moreover, a recent clinical trial has been initiated to evaluate the ability to trace iron oxide-labeled mesenchymal stromal cells (MSC) with MRI after NOGA-guided injection therapy into the myocardium for the treatment of ischemic heart disease [117]. Thus, the ability of USPIO labeled MSC injection to form new heart muscle cells and blood vessels in the myocardium, in order to improve myocardial blood flow and reduce patients’ symptoms, will be analyzed.

#### 2.2.3. Diabetes

A new drug based on the use of GNPs combined with peptide for the treatment of type I diabetes is in its first clinical trial passes [127]. Type 1 diabetes appears when the immune system (body’s own white blood cells) destroys the beta cells that produce insulin in the pancreas. The objective is to develop a treatment that can slow or block this process by switching off the white blood cells causing the damage. In this clinical trial researchers investigate the safety and side effect of C19-A3 GNP, a peptide fragment related to insulin attached to GNPs, as a new therapeutic agent.

#### 2.2.4. Cancer 

In the field of oncology, perfect treatment is one that can be administrated under local anesthetic, can effectively abolish tumor cells, its bystander effect is limited only to surrounding tissues, can be repeatable, and adaptable to future discoveries such as anti-tumor molecular targeting. Magnetic thermoablation, that involves direct injection of MNPs into the tumor, may be able to fulfill these attributes. Magnetic thermoablation uses magnetic field to heat MNPs up to very high temperatures that destroy tumoral cells. In prostate cancer this approach has been investigated in two different ways (i) using MNP thermotherapy alone [128] and (ii) in combination with permanent seed brachytherapy [129]. In both trials, good tolerability and feasibility was shown using the first prototype of an AMF applicator [130]. This innovative approach requires specific tools for thermal monitoring, modeling techniques and quality control based on suitable imaging. Recently, an early phase 1 clinical trial was registered to investigate whether the MNPs remain in their injection site. This is essential to ensure the safety of this system. The consequences of their displacement can be fatal. Firstly, the MNPs could move away from the tumor cells resulting in a poor effectiveness of treatment as targeted cells will not be heated effectively. Secondly, the MNPs can reach sensitive structures around the prostate such as nerves controlling erections, bladder, sphincter muscle controlling urine flow, etc. In this case, the patient could develop serious side effects [131]. In this clinical trial, instead of heating the MNPs, the investigators will use special scans and then surgery, they will determine the exact localization of MNPs. Once determining its location in the injection site, the investigators will then be able to run another study using magnetic thermoablation to treat prostate cancer patients. 

Lyso-thermosensitive liposomal is another system for antitumor targeted drug delivery that is being subjected to several clinical trials, with promising preliminary results. ThermoDox® (Celsion Corporation, Lawrenceville, NJ, USA) is a long-circulating Lyso-Thermosensitive Liposomal Doxorubicin (LTLD) approved for investigational use. ThermoDox® is a heat-activated drug delivery system supplied by IV infusion and facilitates targeted delivery of a cytotoxic drug (DOX) to tumors at temperatures exceeding 40 °C [132]. It was designed to be used in combination with heat-based treatments, such as microwave hyperthermia, high-intensity focused ultrasound (HIFU), or radiofrequency thermal ablation (RFA). The goal is to expand the effective treatment zone in order to capture micrometastases, which are most commonly responsible for post-treatment disease recurrence. In this context, TARDOX is a Phase 1 single center study of the administration of drugs directed by ultrasound in patients with incurable liver tumors. This proof of concept study was designed to prove the safety and feasibility of ThermoDox® unchained by mild hyperthermia induced by ultrasound focused on liver tumors [133,134]. This early phase 1 study demonstrated that the combination of LTLD and non-invasive ultrasound hyperthermia seemed to be clinically achievable, safe, and able to improve intratumoral drug delivery, providing targeted chemo-ablative response in human liver tumors that were intractable by standard chemotherapy [135]. A similar clinical trial is still ongoing for pediatric refractory solid tumors [136]. Other phase 2 and 3 clinical trials are investigating the safety, viability, and effectiveness of ThermoDox® combined with standardized radiofrequency ablation for the treatment of both colon cancer liver metastasis [137] and hepatocellular carcinoma [138]; and microwave hyperthermia in the treatment of recurrent regional breast cancer [139,140]. 

## 3. Conclusions

Thermosensitive materials are very promising for biomedical treatments. However, much work remains to be done to solve its main limitations and facilitate its transfer to the clinic. In this context, one of the most important drawbacks of these nanoparticles is the cleareance and prolonged tissue retention which can cause toxicity. Moreover, some nanoparticles fail to overcome biological barriers end up accumulating in organs outside the target, such as the liver and spleen. This produces a non-specific distribution that negatively affects the diagnosis and treatment of tumors, since when generating hyperthermia, collateral damage in healthy organs cannot be avoided. Furthermore, for the use of MNPs combined with images of multiple modalities to be effective and simplify the non-invasive monitoring of treatments, a dose of MNPs particles necessary to produce the heating should be lowered so as not to cause saturation of the transverse relaxation time of MRI.

After having carried out an exhaustive search on the clinical trials that have been or are being developed on thermosensitive materials, we can conclude that (i) thermosensitive materials, particularly biosensors investigated for analytical and diagnostic applications (e.g., metal-based nanoparticles, thermosensitive polymers, thermoresponsive nanocomposites, and thermochromic dyes) have not reached the clinical trial phase yet. Indeed, all clinical trials consulted focus on the use of ferumoxytol as a current imaging test enhancer except for two newly started trials in September 2018. The first one uses MNPs in the treatment of infertility, and the second one investigates the safety of carbon black and graphene nanoparticles. None of the existing trials take advantage of the thermosensitive characteristics of nanoparticles. (ii) In the field of treatment, the thermosensitive characteristics of nanomaterials are being further exploited especially for the delivery of antitumor drugs. In this regard, ThermoDox® based on lysolipid thermally sensitive liposome technology to encapsulate DOX is the flagship drug. Currently, at the preclinical level, other liposomal formulations such as thermosensitive liposomal nanoparticle (TSLnp) loaded by GEM and paclitaxel (PLX) are under study for theranostic use in solid tumors. TSLnps significantly improved GEM and PLX delivery and enhanced its antitumor activity [50,142]. However, the formulation of Gd-TSLnp needs to be fully optimized to significantly enhance MRI contrast in tumor [50]. Moreover, multiple nano-liposomal systems have been successfully developed for drug delivery for non-tumoral pathologies, such as ocular diseases [143]. Other diseases like IDA, diabetes, and ischemic heart disease are also a target; pathologies are being treated by thermosensitive nanoparticles using USPIOs, fragment-related peptide to GNPs (C19-A3 GNP), and iron oxide-labeled mesenchymal stromal cells, respectively.

The discrepancy existing between the number of papers published on thermosensitive nanomaterials and the number of nanomaterials in clinical use could be due to several factors. On the one hand, there is the relative novelty of this area of research; the first manuscript related to thermosensitive nanoparticles published in PubMed is based on data from 2002, hence the need for a period of time for its validation through clinical trials before its clinical application. On the other hand, the complexity of thermo-nanocarriers prevents their use in delivering drugs. Indeed, reformulating most currently approved drugs, in nanosize form will provide a small improvement in performance that does not compensate for the efforts made by and the costs to pharmaceutical companies. This has already been observed in other areas of research in the field of nanomedicine [144,145]. However, we have no doubt that in the coming years there will be an explosion of clinical trials related to thermosensitive nanomaterials that will surely help to improve current treatments and above all will impact on patients’ quality of life and life expectancy.

## Figures and Tables

**Table 1:** New TSLs systems developed for cancer treatment and diagnosis.

Type of TSLs	Drug	Improvement	Use	Ref.
Liposome formulations of HTLC	HTLC	Treatment of cervical carcinoma	Treatment	[56]
Liposomal formulations with drug	GEM DOX	Increased drug plasma half-life	Treatment	[55] [65]
Multi-modal thermo-sensitive polymer-modified liposomes (MTPLs)	MnSO4, rhodamine, DOX	Monitor drug delivery in cancer therapy	Diagnosis	[66]
Liposomal ICG	ICG	Deliver ICG as the NIR in the treatment of triple negative breast cancer	Treatment	[67]
TSLs loaded with drug and fullerene decorated with IONs	ICG, DOX	Multifunctional TSLs showing radiofrequency release and MRI for tumor therapy	Diagnosis and treatment	[60]
TSLs loaded with drugs and mesoporous silica nanoparticles	ICG, DOX	Multifunctional nanoplatform for integrate diagnosis and treatment (photodynamic therapy and PTT) for cancer	Diagnosis and treatment	[68]

**Table 2:** Current clinical trials of thermosensitive nanomaterials for diagnosis of various diseases available on clinicaltrials.gov up to November 2018.

Pathology	Interventions	ClinicalTrials ID	Phase	Date (First–Last Posted)	Ref.
Blood Biomarkers Vasodilation Blood Clotting Lung Function Healthy Volunteers	- Diesel exhaust particulate	NCT03659864	Not applicable	September 2018–still active	[69]
	- Carbon nanoparticles				
	- Small graphene oxide				
	- Ultrasmall graphene oxide				
Metastatic and Primary Brain Neoplasm	- Ferumoxytol	NCT00659126	Phase 2	2018–still active	[74]
	- 3 Tesla MRI				
	- Dynamic Contrast-Enhanced MRI				
Head and Neck Cancer	- Ferumoxytol	NCT01895829	Early Phase	2013–still active	[75]
	- MRI				
Solid Tumors	Ferumoxytol followed by MM-398	NCT01770353	Phase 1	2013–still active	[76]
ER/PR Positive Breast Cancer					
Active Brain Metastasis					
Rectal Cancer (Stage III) Esophageal Cancer (Stage II–III)	- Ferumoxytol - MRI - PET/CT	NCT03280277	Phase 2	2017–still active	[77]
Cancer of Lymph Node	- Feraheme	NCT01815333	Not applicable	2013–still active	[78]
	- MRI				
Prostate Cancer	Drug: Ferumoxytol	NCT01296139	Phase 1	2011–2018	[79]
Prostate Cancer Bladder Cancer Kidney Cancer	- Ferumoxytol - MRI	NCT02141490	Phase 2	2014–still active	[80]
Breast Cancer	- Ferumoxytol	NCT00087347	Not applicable	2013–still active	[81]
Prostate Cancer	- MRI				
Soft Tissue Sarcoma	- Ferumoxytol	NCT00978562	Not applicable	2017–still active	[82]
Childhood Brain Neoplasm	- MRI				
Brain Injury	- Ferumoxytol	NCT02452216	Early Phase 1	2015–2017	[83]
CNS: Degenerative and Infectious Disorder	- MRI				
Childhood Brain Neoplasm	- Ferumoxytol	NCT03179449	Early Phase 1	2017–still active	[84]
	- MRI				
CNS Brain Neoplasm	- Ferumoxytol - 3 Tesla MRI - Dynamic MRI	NCT02857218 NCT00103038	Not applicable	2005–still active	[107] [85]
Bone Cancer Chondrosarcoma Ewing’s Sarcoma	- Feraheme - MRI	NCT01336803	Not applicable	2011–still active	[87]
Glioblastoma	- Pembrolizumab	NCT03347617	Phase 2	2017–still active	[89]
Malignant Primary and metastatic Brain Neoplasm	- Ferumoxytol				
Melanoma	- MRI				
Adult Brain Glioblastoma	- Gadolinium	NCT00660543	Not applicable	2016–2017	[90]
	- Ferumoxytol				
	- MRI				
Brain Neoplasms	Ferumoxytol	NCT00769093	Phase 1	2008–2017	[91]
Myocardial Infarction	- Ferumoxytol	NCT01323296	Not applicable	2011–2014	[92]
	- MRI				
Hereditary Hemorrhagic Telangiectasia	Feraheme MRI/MRA	NCT02977637	Phase 1	2016–still active	[93]
Diabetes Mellitus, Type 1	- Ferumoxytol	NCT00585936	Not applicable	2008–2011	[94]
	- MRI				
Type 1 Diabetes	- Ferumoxytol	NCT01521520	Not applicable	2012–still active	[95]
	- MRI				
Renal Transplant Rejection	- Feraheme	NCT02006108	Not applicable	2017–2018	[96]
	- MRI-GE Healthcare 3 Tesla magnet				
Osteonecrosis	- Ferumoxytol	NCT02893293	Phase 4	2015–still active	[97]
	- MRI				
CNS Neoplasm	- Ferumoxytol	NCT03270059	Phase 2	2017–still active	[98]
	- Gadolinium				
	- MRI				
MS	- Ferumoxytol	NCT02511028	Early Phase 1	2015–still active	[99]
	- MRI				
Epilepsy	- Ferumoxytol injection after focal epileptic seizure	NCT02084303	Not applicable	2014–2018	[100]
	- MRI				
HIV Dementia	- Ferumoxytol	NCT01665846	Phase 1	2012–2018	[101]
AIDS Dementia Complex	- Ferumoxytol	NCT02678767	2016-2017	Phase 2	[102]
	- MRI				
Peripheral Arterial Disease	- Ferumoxytol	NCT00707876	Phase 2	2008–still active	[104]
	- MRI				
Cerebrovascular Disorders	Quantitative MRI	NCT03266848	Not applicable	2017–still active	[105]
Infertility	MNP Sperm Separation for ICSI Cycles	NCT03666364	Not applicable	September 2018–still active	[106]
Acute Coronary Syndrome	Immunomagnetic reduction by MNPs	NCT02226523	Not applicable	2014–still active	[108]
Lung Carcinoma	- Pembrolizumab	NCT03325166	Phase 2	2017–still active	[109]
	- Ferumoxytol				
	- MRI				
Childhood Brain Neoplasm	- Ferumoxytol	NCT03234309	Phase 2	2017–still active	[110]
	- MRI				
Nervous System Diseases	- Ferumoxytol	NCT00659776	Phase 2	2008–2012	[111]
	- MRI				
Cardiac Transplant Cardiac Sarcoid Myocarditis	- Ferumoxytol	NCT02319278	Phase 2	2014–2017	[112]
	- MRI		Phase 3		
Pediatric Congenital Heart Disease	- Ferumoxytol	NCT02752191	Phase 4	2016–still active	[113]
	- Gadofosveset				
Coronary Artery Disease	- Ferumoxytol	NCT02954510	Phase 3	2016–still active	[114]
	- MRI				
- Coronary Artery Disease - Atherosclerosis	- Stenting and micro -infusion of GNPs	NCT01436123	Phase 1	2011–2015	[115]
	- Implantation of everolimus-eluting stent				
Atherosclerosis	- Ferumoxytol - MRI scan	NCT01674257	Not applicable	2012–2013	[116]
Stroke	- Radiation: 18f-Fluoride PET/CT - Radiation: 18F-Flurodeoxyglucose PET/CT				
Ischemic Heart Disease	Iron oxide-labeled mesenchymal stromal	NCT03651791	Phase 1	August 2018–still active	[117]
	MRI				

**Table 3:** Current clinical trials of thermosensitive nanomaterials for treatment of various diseases available on clinicaltrials.gov until November 2018.

Pathology	Interventions	ClinicalTrials ID	Phase	Date (First–Last Posted)	Ref.
CKD	- Ferumoxytol	NCT02997046	Phase 4	2016‒still active	[104]
	- MRA				
Ischemic Heart Disease	- Iron oxide-labeled mesenchymal stromal cells	NCT03651791	Phase 1	August 2018‒still active	[117]
	- MRI				
IDA	- Ferumoxytol	NCT03619850	Phase 3	August 2018‒still active	[120]
Pediatric CKD	- Oral Iron				
IDA	Intravenous and Oral Iron	NCT03657433	Phase 3	September 2018	[121]
Pregnancy					
IDA	- Ferumoxytol	NCT01227616	Phase 4	2010‒2017	[125]
CKD	- Iron Sucrose				
- Stable Angina - Heart Failure - Atherosclerosis - Multivessel Coronary Artery Disease	- GNPs - Iron-bearing nanoparticles - Stenting	NCT01270139	Not applicable	2012‒2017	[126]
Type 1 Diabetes	- C19-A3 GNP (peptide fragment related to insulin attached to GNPs)	NCT02837094	Phase 1	2016‒still active	[127]
Prostate Cancer	MNPs Injection	NCT02033447	Early Phase 1	2013‒2017	[131]
Liver Tumor	- ThermoDox®	NCT02181075	Phase 1	2014‒2017	[133]
	- Magnetic resonance high intensity focused ultrasound				
Pediatric Cancer Solid Tumors	- ThermoDox®	NCT02536183	Phase 1	2015‒still active	[136]
	- MRI				
	- High-intensity focused ultrasound				
Colon Cancer	- ThermoDox®	NCT01464593	Phase 2	2011‒2016	[137]
Liver Metastasis	- Radiofrequency ablation				
Hepatocellular Carcinoma	- ThermoDox®	NCT02112656	Phase 3	2014‒still active	[138]
	- Radiofrequency ablation				
Breast Cancer	- ThermoDox®	NCT00826085	Phase 2	2009‒2017	[139]
	- Microwave hyperthermia				
Breast Cancer	- ThermoDox®	NCT03749850	Phase 1	November 2018‒still active	[140]
	- MR-HIFU induced hyperthermia				
	- Cyclophosphamide				
- IDA	- Ferumoxytol	NCT01155388	Phase 3	2017‒still active	[141]
- Non-dialysis-dependent CKD	- Oral Iron				
